# PET/CT for differentiating between tuberculous peritonitis and peritoneal carcinomatosis: The parietal peritoneum

**DOI:** 10.1097/MD.0000000000005867

**Published:** 2017-01-13

**Authors:** Shao-Bo Wang, Yun-Hai Ji, Hu-Bing Wu, Quan-Shi Wang, Wen-Lan Zhou, Liang Lv, Tao Shou, Jing Hu

**Affiliations:** a PET/CT Center, the First People's Hospital of Yunnan Province, Kunming; b NanFang PET Center, Nanfang Hospital, Southern Medical University, Guangzhou; c Department of Radiology; d Department of Medical Oncology, The First People's Hospital of Yunnan Province, Kunming, China.

**Keywords:** diagnosis, peritoneal carcinomatosis, positron emission tomography/computed tomography, scintigraphic patterns, tuberculous peritonitis

## Abstract

**Objectives:**

Tuberculous peritonitis (TBP) mimics peritoneal carcinomatosis (PC). We aimed to investigate the discriminative use of PET/CT findings in the parietal peritoneum.

**Materials and Methods:**

Parietal peritoneal PET/CT findings from 76 patients with TBP (n = 25) and PC (n = 51) were retrospectively reviewed. The lesion locations were noted as right subdiaphragmatic, left subdiaphragmatic, right paracolic gutters, left paracolic gutters, and pelvic regions. The distribution characteristic consisted of a dominant distribution in the pelvic and/or right subdiaphragmatic region (susceptible area for peritoneal implantation, SAPI) (SAPI distribution), a dominant distribution in the remaining regions (less-susceptible area for peritoneal implantation, LSAPI) (LSAPI distribution), or a uniform distribution. PET morphological patterns were classified as F18-fluorodeoxyglucose (^18^F-FDG) uptake in a long beaded line (string-of-beads ^18^F-FDG uptake) or in a cluster (clustered ^18^F-FDG uptake) or focal ^18^F-FDG uptake. CT patterns included smooth uniform thickening, irregular thickening, or nodules.

**Results:**

More common findings in the parietal peritoneum corresponding to TBP as opposed to PC were (a) ≥4 involved regions (80.0% vs 19.6%), (b) uniform distribution (72.0% vs 5.9%), (c) string-of-beads ^18^F-FDG uptake (76.0% vs 7.8%), and (d) smooth uniform thickening (60.0% vs 7.8%) (all *P* < 0.001), whereas more frequent findings in PC compared with TBP were (a) SAPI distribution (78.4% vs 28.0%), (b) clustered ^18^F-FDG uptake (56.9% vs 20.0%), (c) focal ^18^F-FDG uptake (21.6% vs 4.0%), (d) irregular thickening (51.0% vs 12.0%), and (e) nodules (21.6% vs 4.0%) (*P* < 0.001, *P* < 0.05, *P* > 0.05, *P* < 0.05, *P* > 0.05, respectively).

**Conclusion:**

Our data show that PET/CT findings in the parietal peritoneum are useful for differentiating between TBP and PC.

## 1 Introduction

Tuberculosis (TB) remains a significant cause of morbidity and mortality in developing countries. Tuberculous peritonitis (TBP) is a rare form of extrapulmonary TB; its reported incidence varies from 0.1% to 0.7% among all forms of TB worldwide, and delay in treatment initiation can lead to high mortality.^[1]^

Unfortunately, it is challenging to differentiate TBP from peritoneal carcinomatosis (PC) because the clinical manifestations and conventional laboratory tests of TBP and PC frequently overlap.^[2,3]^ Ascites cytology has low positive detection rates.^[2,4]^ Ascites adenosine deaminase (ADA) measurement is helpful in the differential diagnosis of TBP and PC; however, ADA is a nonspecific inflammatory and immune response marker for TB, and false negative results or false positive results are frequent in various clinical situations, such as TBP in patients with liver cirrhosis or HIV infection, PC, and bacterial peritonitis.^[3,5]^ A mycobacterial culture of ascitic fluid is problematic because tuberculous peritonitis-associated mortality is high among patients waiting for the results.^[6]^ A T-cell-based enzyme-linked immunospot assay (T-SPOT.TB) is useful for the diagnosis of latent tuberculosis infection but has a limited role in individuals with a previous history of TB.^[7,8]^ Although peritoneal biopsy has a higher diagnostic accuracy, it is limited due to its invasiveness, sampling error, and complications (e.g., bleeding, infection, and bowel perforation).^[4,9,10]^

Thus, medical imaging is crucial for differentiating TBP from PC. Specifically, peritoneal CT findings are valuable for this differential diagnosis but still yield an undetermined diagnosis or even a misdiagnosis.^[11–14]^ In contrast, F18-fluorodeoxyglucose(^18^F-FDG) PET has a high sensitivity for detecting peritoneal lesions because ^18^F-FDG PET can clearly detect harboring lesions as high-uptake foci.^[15–17]^ Furthermore, malignant and benign peritoneal involvement may manifest as various imaging patterns on ^18^F-FDG PET, and awareness of the disease-specific characteristics is important to optimize diagnostic accuracy.^[18]^^18^F-PET/CT is performed for TBP patients to further diagnose after conventional clinical examinations present undetermined diagnoses or suspected PC, particularly in countries having medium-to-high TB burdens^[4,19–22]^ and occasionally in other countries.^[23,24]^

^18^F-FDG PET/CT is used in the evaluation of peritoneal diseases of undetermined causes, but special attention should be paid to TBP mimicking PC.^[4,19]^ However, until recently, there has been a lack of comparison studies that discuss how to differentiate between TBP and PC using ^18^F-FDG PET/CT.^[15,16,22]^ The present study aimed to investigate discriminative PET/CT findings in the parietal peritoneum between the 2 entities.

## 2 Materials and methods

### 2.1 Study cohort

This study was approved by the Institutional Review Board of Nanfang Hospital, Southern Medical University. Because of the retrospective nature of the study, the requirement of informed consent was waived.

From July 2004 to January 2015, 76 consecutive patients with TBP (n = 25) and PC (n = 51) who underwent ^18^F-FDG PET/CT before therapy were retrospectively enrolled. TBP was confirmed by peritoneal pathology using surgery (n = 10) and laparoscopy (n = 2) or by diagnostic anti-TB therapy and at least 12 months of clinical follow-up (n = 13). PC was confirmed by peritoneal pathology using surgery (n = 13) and laparoscopy (n = 18) or by ascites cytology (n = 20). No patients had both TB and malignancy concomitantly. The clinical characteristics are shown in Table 1.

**Table 1:**
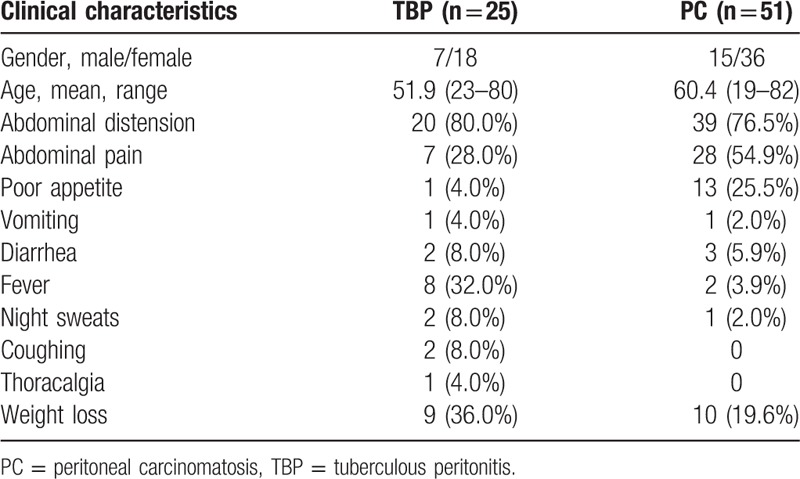
The clinical characteristics of tuberculous peritonitis (TBP) and peritoneal carcinomatosis (PC) patients.

Among the 25 TBP patients, concomitant TB at other sites was present in the ovary (n = 9), mediastinal lymph node (n = 8), lung (n = 6), ileocecum (n = 3), endometrium (n = 1), and fallopian tube (n = 1).

Among the 51 PC patients, the primary cancer was present in the ovary (n = 19), stomach (n = 9), colon and rectum (n = 7), bile duct (n = 3), liver (n = 1), fallopian tube (n = 1), and endometrium (n = 1); the primary cancer was undetermined in 10 cases.

### 2.2 Image acquisition

Eleven TBP patients and 30 PC patients underwent a Discovery LS PET/CT Scanner (GE Healthcare, Waukesha, WI). Fourteen TBP patients and 21 PC patients underwent a Biograph mCTx PET/CT Scanner (Siemens AG, Munich, Germany). The ^18^F was produced in a PET trace cyclotron (GE Healthcare). The ^18^F-FDG was automatically synthesized in a chemical synthesis module (Beijing PET Biotechnology Co., Ltd., China) with a radiochemical purity >95%. After fasting for more than 6 h in a calm state, the patient was intravenously injected with 0.15 mCi (5.5 MBq)/kg of ^18^F-FDG followed by lying in a dark room for approximately 1 h. PET and nonenhanced CT imaging was performed after emptying the urinary bladder. Scanning was performed from the middle femur to the cranial vault. The PET images were reconstructed according to an iterative ordered subset expectation maximization method. The reconstruction thicknesses of the CT images were 4.25 mm and 3.0 mm, and the PET and CT images were individually transferred to Xeleris (GE Healthcare) or Syngo MMWP (Siemens) workstations, respectively, to display frame-on-frame fusion images.

### 2.3 Imaging analysis

All of the following PET/CT findings were separately reviewed by 2 nuclear medicine specialists who were blinded to all laboratory data and other imaging examinations. If interpretive disagreements occurred, the final reports were decided by a third nuclear medicine specialist.

On each PET image, the region of interest (ROI) was drawn along the margin of the lesion to measure the standardized uptake value (SUV). The SUV is commonly used as a relative measure of FDG uptake. The basic expression for SUV is 
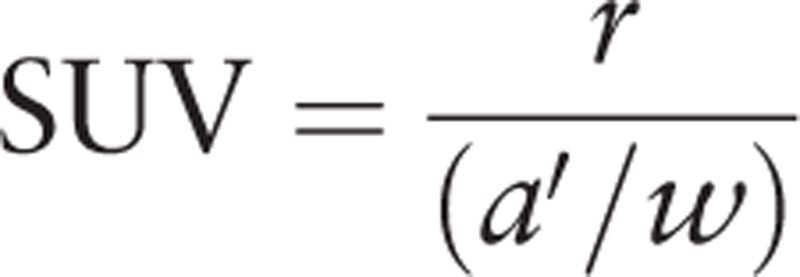


where *r* is the radioactivity activity concentration (kBq/mL) measured by the PET scanner within an ROI, *a*′ is the decay-corrected amount of injected radiolabeled FDG (kBq), and *w* is the weight of the patient (g), which is used a surrogate for a distribution volume of trace.^[25]^ Because the maximum of SUV (SUV_max_) has a significantly improved reproducibility compared to the mean SUV (SUV_mean_),^[25]^ the SUV_max_ was used in this study. The confirmation of parietal peritoneal lesions was based on the requisite exclusion of physiologic bowel activity, retained urinary activity, misregistration artifacts, attenuation correction artifacts, blooming from highly hypermetabolic lesions, respiration artifacts at the hemidiaphragm, and surrounding abnormalities from the viscera, visceral peritoneum, omentum, mesentery, and other peritoneal structures. The location of the parietal peritoneal lesions was noted as the right subdiaphragmatic, left subdiaphragmatic, right paracolic gutter, left paracolic gutter, and pelvic regions.^[14,26]^

Because the pelvic and right subdiaphragmatic regions of the parietal peritoneum are susceptible areas for peritoneal implantation (SAPI),whereas the remaining regions are less-susceptible areas for peritoneal implantation (LSAPI),^[26–28]^ the distribution of the parietal peritoneum lesions was classified as the SAPI distribution, in which the lesions were completely or primarily localized in the pelvic and/or right subdiaphragmatic region; the LSAPI distribution, in which the lesions were completely or primarily localized in the remaining regions; or the uniform distribution, in which the lesions were uniformly distributed in the susceptible and less-susceptible areas.

**Figure 1:**
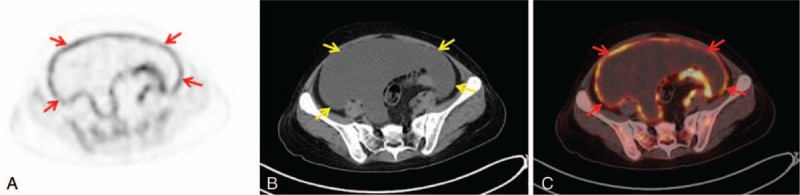
A 37-year-old man with tuberculous peritonitis: string-of-beads ^18^F-FDG uptake in the parietal peritoneum (red arrow) on PET and combined PET/CT axial imaging (A and C); smooth uniform thickening (yellow arrow) on axial CT imaging (B). CT = computed tomography,^ 18^F-FDG = F18-fluorodeoxyglucose, PET = positron emission tomography.

**Figure 2:**
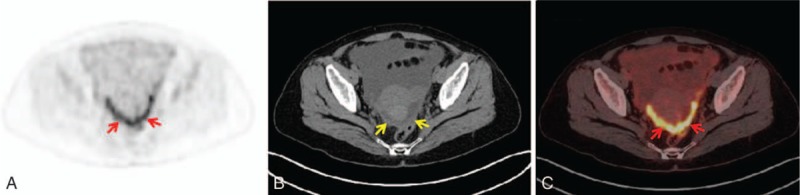
A 51-year-old woman with peritoneal carcinomatosis of gastric cancer: clustered ^18^F-FDG uptake in the parietal peritoneum (red arrow) on PET and combined PET/CT axial imaging (A and C); irregular thickening in the pelvic region (yellow arrow) on CT axial imaging (B). CT = computed tomography,^ 18^F-FDG = F18-fluorodeoxyglucose, PET = positron emission tomography.

**Figure 3:**
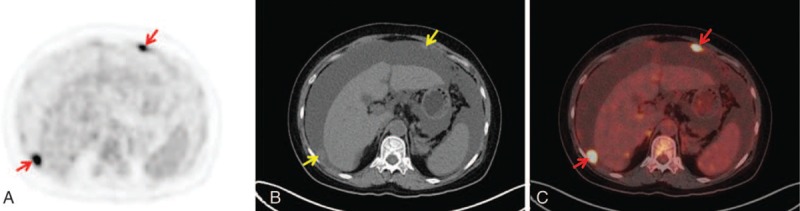
A 64-year-old woman with peritoneal carcinomatosis of colon cancer: focal ^18^F-FDG uptake in the parietal peritoneum (red arrow) on PET and combined PET/CT axial imaging (A and C); nodular abnormalities (yellow arrow) shown on CT axial imaging (B). CT = computed tomography, ^18^F-FDG = F18-fluorodeoxyglucose, PET = positron emission tomography.

According to the continuity observed among the ^18^F-FDG-avid lesions in the sectional PET images, the morphological patterns included the following: (1) lesions continuously disseminated beyond a single region showing^18^F-FDG uptake in a long beaded line (string-of-beads ^18^F-FDG uptake) (Figs. 1A and C); (2) lesions continuously disseminated in a single region showing clustered ^18^F-FDG uptake (Figs. 2A and C); or (3) lesion(s) discontinuously disseminated showing isolated or discrete ^18^F-FDG uptake (focal ^18^F-FDG uptake) (Figs. 3A and C). If multiple patterns coexisted, a string-of-beads accompanied by a clustered and/or focal ^18^F-FDG uptake was considered a string-of-beads ^18^F-FDG uptake, whereas a clustered ^18^F-FDG uptake accompanied by a focal ^18^F-FDG uptake was considered a clustered ^18^F-FDG uptake.

On the CT imaging, the CT findings were classified as smooth uniform thickening (Fig. 1B), irregular thickening (Fig. 2B), or nodules (Fig. 3B).^[14,29]^

### 2.4 Statistical analysis

The SUV_max_ and the number of regions with parietal peritoneum involvement were compared between the 2 entities using a receiver operating characteristic (ROC) curve analysis. The lesion distributions and the morphological presentations were compared between these 2 entities using the χ^2^ test, the continuity correction χ^2^ test, or Fisher's exact test. The data were analyzed using MedCalc, version 13.0.0.0 (MedCalc Software, Ostend, Belgium). If *P* < 0.05, the difference was considered statistically significant.

## 3 Results

### 3.1 SUV_max_

The SUV_max_ calculations for the peritoneal lesions with the most significant ^18^F-FDG uptake between TBP and PC were 7.8 ± 3.3 (2.8–14.8) and 7.0 ± 3.7 (2.0–15.5), respectively. The area under ROC curve (AUC) was 0.591 (*P* = 0.174).

### 3.2 Distribution range

The involvement of the parietal peritoneum was noted in 25 (100%) TBP patients and 44 (86.3%) PC using PET, 19 (76.0%) TBP patients and 41 (80.4%) PC patients using CT, and 25 (100%) TBP patients and 44 (86.3%) PC patients using both PET and CT.

**Figure 4:**
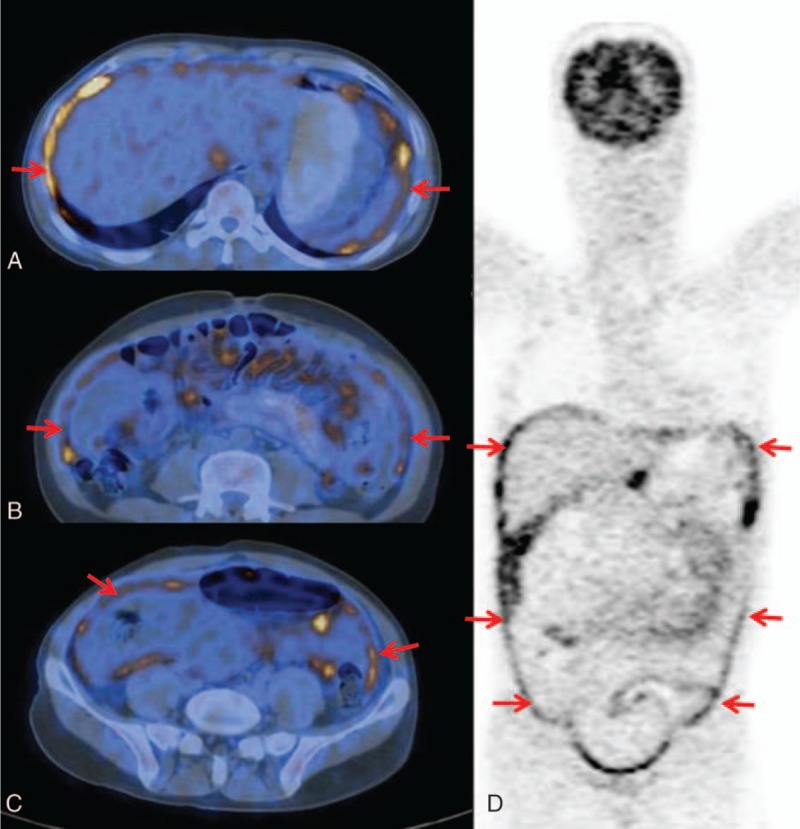
A 37-year-old man with tuberculous peritonitis. Diffuse involvement and uniform distribution in the parietal peritoneum: string-of-beads ^18^F-FDG uptake (red arrow) and uniform distribution in the bilateral subdiaphragmatic regions (A), the bilateral paracolic gutter regions (B), and the pelvic region (C) on combined PET/CT axial imaging; string-of-beads ^18^F-FDG uptake (red arrow) in all 5 regions of the parietal peritoneum on whole-body PET coronal-sectional imaging (D). CT = computed tomography, ^18^F-FDG = F18-fluorodeoxyglucose, PET = positron emission tomography.

**Figure 5:**
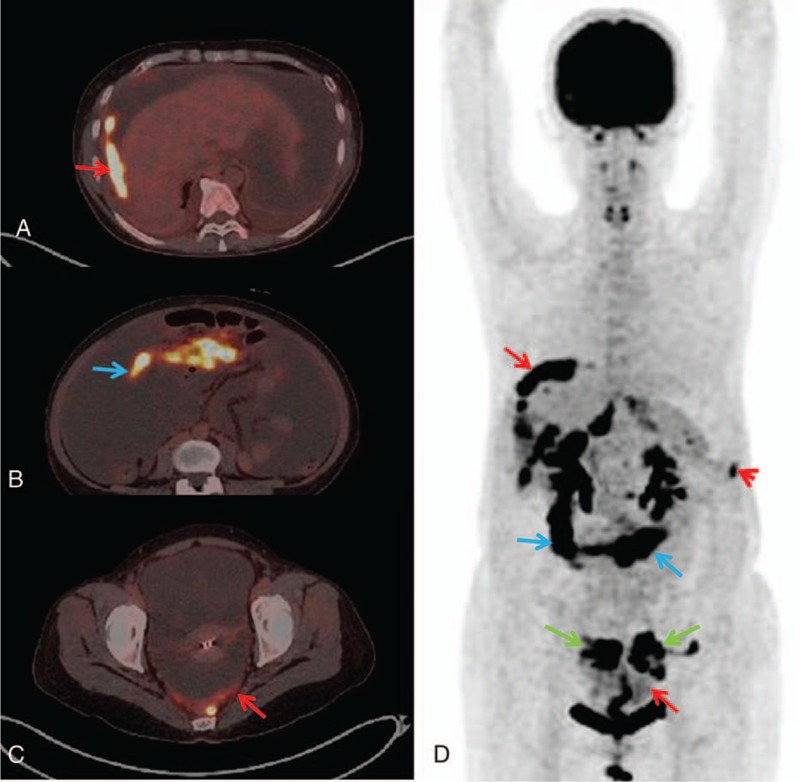
A 46-year-old woman with peritoneal carcinomatosis from ovarian cancer. Dominant distribution in the pelvic and right subdiaphragmatic region of the parietal peritoneum (the susceptible area for peritoneal implantation, SAPI) (SAPI distribution): clustered ^18^F-FDG uptake (red arrow) in the right subdiaphragmatic region (A) and the pelvic region (C) and negative findings in the bilateral paracolic gutter regions (B) on combined PET/CT axial imaging; focal ^18^F-FDG uptake (red arrowhead) in the left subdiaphragmatic region on PET maximum intensity projection (MIP) imaging (D); mass-like ^18^F-FDG uptake in the bilateral ovaries (green arrow) and the greater omentum (blue arrow) (D). CT = computed tomography, ^18^F-FDG = F18-fluorodeoxyglucose, MIP = maximum intensity projection, PET = positron emission tomography, SAPI = susceptible area for peritoneal implantation.

Among the 5 regions for locating the parietal peritoneal lesions, the median (interquartile range) of regions with parietal peritoneal involvement in TBP and PC patients was 5 (1) and 2 (2), respectively, with an AUC = 0.863 and a cut-off≥4. In addition, ≥4 involved regions in the parietal peritoneum occurred in 20 (80.0%) TBP patients and 10 (19.6%) PC patients (*P* < 0.001) (Figs. 4 and 5).

### 3.3 Distribution characteristics

Table 2 shows the distributions of the parietal peritoneal lesions. The SAPI distribution in the TBP patients (28.0%) occurred less commonly compared with the PC patients (78.4%), *P* < 0.001 (Fig. 5). The LSAPI distribution was exclusively noted in PC patients (2.0%), *P* > 0.05. The uniform distribution in TBP patients (72.0%) occurred more frequently than in PC patients (5.9%), *P* < 0.001 (Fig. 4).

**Table 2:**
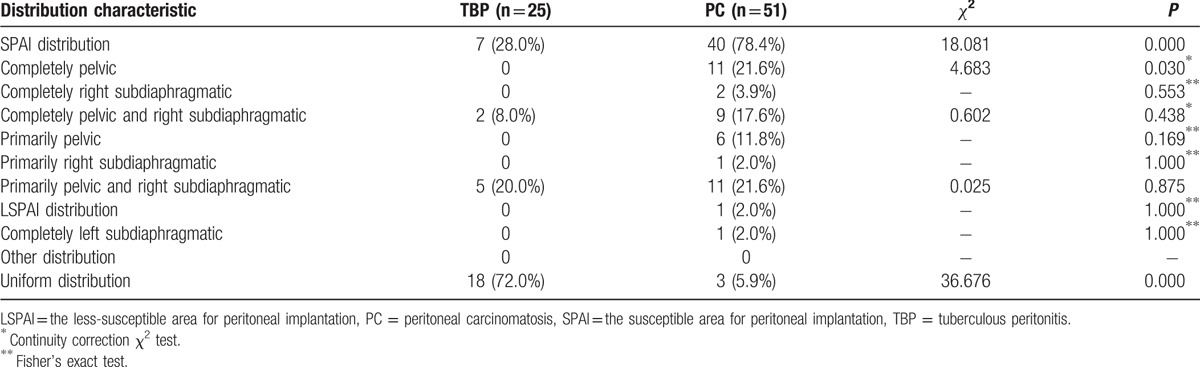
Distribution characteristic of the parietal peritoneum lesions in tuberculous peritonitis (TBP) and peritoneal carcinomatosis (PC) patients.

### 3.4 Morphological patterns

Table 3 shows the morphological patterns of the parietal peritoneal lesions. With regard to PET patterns, string-of-beads^18^F-FDG uptake (string-of-beads sign) occurred more frequently in TBP patients (76.0%) than in PC patients (7.8%), *P* < 0.001. Clustered ^18^F-FDG uptake (clustered sign) was observed in 20.0% of TBP patients and 56.9% of PC patients, *P* < 0.05. Focal ^18^F-FDG uptake was noted in 4.0% of TBP patients and 21.6% of PC patients, *P*>0.05.

**Table 3:**
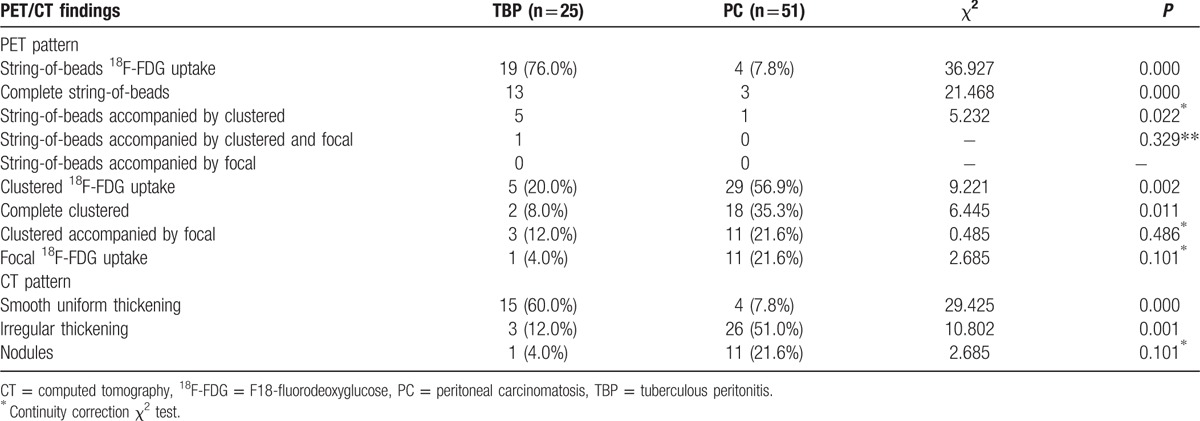
Morphological findings of the parietal peritoneum in tuberculous peritonitis (TBP) and peritoneal carcinomatosis (PC) patients.

With regard to CT patterns, smooth uniform thickening in the parietal peritoneum occurred more frequently in TBP patients (60.0%) than in PC patients (7.8%), *P* < 0.001. Irregular thickening in TBP patients (12.0%) was less common than in PC patients (51.0%), *P* < 0.05. Nodules were noted in TBP patients (4.0%) and PC patients (21.6%), *P* > 0.05.

### 3.5 Diagnostic performance

When TBP was diagnosed based on one of the differential findings between the 2 entities, the sensitivities and specificities were 80.0% and 80.4% (≥4 involved regions in the parietal peritoneum), 72.0% and 94.1% (uniform distribution), 76.0% and 92.2% (string-of-beads sign), and 60.0% and 92.2% (smooth uniform thickening) (all *P* < 0.001).

For the diagnosis of PC, the sensitivities and specificities were 78.4% and 72.0% (SAPI distribution) (*P* < 0.001), 56.9% and 80.0% (clustered sign) (*P* < 0.05), and 51.0% and 88.0% (irregular thickening) (*P* < 0.05).

## 4 Discussion

This study preliminarily reported the difference in ^18^F-FDG PET/CT findings between TBP and PC, including glucose metabolism, distribution range, distribution characteristics, and morphological patterns of peritoneal lesions.

As a semiquantitative indicator for reflecting glucose metabolism in lesions, SUV_max_ is considered an important indicator for the differential diagnosis of benign versus malignant peritoneal lesions.^[4,15]^ However, this study indicated that SUV_max_ did not reveal a significant difference between TBP and PC (*P* > 0.05). TB lesions contain a large number of epithelioid cells, lymphocytes, and Langerhans cells that have a high expression of glucose transporter 1 (Glut-1) and Glut-3, which induced high ^18^F-FDG uptake.^[4]^ This might be one of the main reasons that published studies have reported that TBP mimics PC on ^18^F-FDG PET/CT when attempting to differentiate between the 2 entities.^[4,21–23,30–32]^

Shimamoto et al^[22]^ proposed that diffuse peritoneal uptake of ^18^F-FDG may be indicative of TBP rather than PC. Other case reports also described diffuse ^18^F-FDG uptake in the peritonea of TBP patients.^[21,23,31,32]^ Conversely, the most frequent pattern was abnormally intense focal ^18^F-FDG uptake, although diffuse and focal ^18^F-FDG uptake in the peritoneum was observed in PC patients.^[15,16]^

This study indicated that the number of regions with parietal peritoneal involvement was significantly higher in TBP patients than in PC patients (*P* < 0.001) and that a cut-off of ≥4 involved regions (extensive involvement) was a significant indicator for diagnosis of TBP with 80.0% sensitivity and 80.4% specificity.

Other publications also indicate that peritoneal lesions exhibit a more extensive range of distribution in TBP patients than in PC patients. Peritoneal implantation through ascites has been considered the most common route in PC patients.^[33,34]^ The distribution of peritoneal implantation through ascites can be restricted by gravity, negative pressure in the subdiaphragmatic space, intestinal peristalsis, and the anatomic features of the abdominal compartment.^[35,36]^ The hematogenous spread of *Mycobacterium tuberculosis* has been considered the most common route in TBP patients,^[37–39]^ which suggests that the spread of the lesions is not restricted by the anatomic structure of the peritoneum.

With the exception of the greater omentum, the sites most commonly involved in peritoneal implants were the right subdiaphragm and the pelvis.^[26]^ The pelvic region (including the pouch of Douglas in women and the retrovesical space in men) is the lowest point of the abdominal cavity in the standing position. This area had a high incidence of lesion implantation through ascites. Although the paracolic sulcus is the lowest point in the reclining position of the human body, the right subdiaphragmatic space is the area where ascites most easily accumulates when in a reclining position due to the influences of intrathoracic negative pressure. The main reason that ascites does not easily accumulate in the left subdiaphragmatic space is restriction by the left phrenicocolic ligament and hepatic falciform ligament. Another reason is that the left paracolic sulcus is shallower than the right paracolic sulcus; thus, ascites can easily reach the right subdiaphragmatic region through the right paracolic sulcus. Therefore, the right subdiaphragmatic region is another area that facilitates the colonization of implanted lesions through ascites.^[27,28]^

This study showed that parietal peritoneal lesions, completely or primarily localized in the pelvic and/or right subdiaphragmatic regions (SAPI distribution), were significant indicators of PC, with 78.4% sensitivity and 72.0% specificity, and that a uniform distribution in the susceptible and less-susceptible areas for peritoneal implantation (uniform distribution) was a significant indicator of TBP, with 72.0% sensitivity and 94.1% specificity (both *P* < 0.001). These results also support the notions that the peritoneal lesion spread of PC is commonly restricted by the anatomical features of the abdominal cavity, that intraperitoneal implantation through ascites might be the most common route of PC, with the peritoneal lesion distribution perhaps differing according to the site of primary cancer, and that hematogenous spread is the most common route for TBP.

Different examination methods demand different diagnostic criteria. Visual laparoscopic diagnosis of TBP was based on the presence of multiple yellowish-white miliary tubercles of uniform size (usually < 5 mm) on the visceral and parietal peritonea. PC was diagnosed by the presence of large nodules (1 to 5 cm in diameter) on the parietal peritoneum, omentum, falciform ligament, or liver surface.^[10]^ CT diagnosis of the 2 entities includes 3 different patterns in the parietal peritoneum: smooth uniform thickening, irregular thickening, and nodules.^[14,29]^ This study also demonstrated that smooth uniform thickening was a significant predictor of TBP, with 60.0% sensitivity and 92.2% specificity, whereas irregular thickening was a significant predictor of PC, with 51.0% sensitivity and 88.0% specificity (both *P* < 0.05).

The morphological changes observed in PET differ from those observed in laparoscopy and CT. For example, the finding of multiple miliary nodules in the parietal peritoneum under laparoscopy is a characteristic presentation of TBP. Although these multiple miliary nodules can be detected, their size is too small (usually <5 mm) to identify their nodular features by CT, which yields a CT pattern of smooth uniform thickening.^[14,29]^ For PET imaging, blooming from these miliary lesions with high hypermetabolism causes an observed lesion size that is larger than its actual size. Consequently, these nodular characteristics identified by PET do not exhibit a smooth uniform presentation (Fig. 1).

In this study, parietal peritoneal involvement was classified into 3 distinct PET patterns: string-of-beads, clustered, and focal ^18^F-FDG uptake. These patterns were differentially observed in TBP and PC patients. The string-of-beads sign was a significant indicator of TBP, with 76.0% sensitivity and 92.2% specificity (*P* < 0.001), and the clustered sign was a significant indicator of PC, with 56.9% sensitivity and 80.0% specificity (*P* < 0.05).

In conclusion, this study has established some basic ^18^F-FDG PET/CT features in the parietal peritoneum for differentiating between TBP and PC. Extensive involvement, a uniform distribution, string-of-beads sign, and smooth uniform thickening might be significant differential features of TBP. A SAPI distribution, clustered sign, and irregular thickening might be significant differential features of PC. However, further investigation with a large patient enrollment is warranted to confirm our findings.
